# Milk Protein Fractionation by Means of Spiral-Wound Microfiltration Membranes: Effect of the Pressure Adjustment Mode and Temperature on Flux and Protein Permeation

**DOI:** 10.3390/foods8060180

**Published:** 2019-05-28

**Authors:** Martin Hartinger, Hans-Jürgen Heidebrecht, Simon Schiffer, Joseph Dumpler, Ulrich Kulozik

**Affiliations:** 1 Chair of Food and Bioprocess Engineering, Technical University of Munich, 85354 Freising, Germany; hans-juergen.heidebrecht@tum.de (H.-J.H.); simon.schiffer@tum.de (S.S.); Joseph.Dumpler@tum.de (J.D.); ulrich.kulozik@tum.de (U.K.); 2 Department of Food Science, Cornell University, Ithaca, NY 14853-5701, USA

**Keywords:** microfiltration, MF, SWM, skim milk, deposit layer history, deposit layer formation, mode of Δp_TM_ increase, temperature

## Abstract

Protein fractionation by means of microfiltration (MF) is significantly affected by fouling, especially when spiral-wound membranes (SWMs) are used. We investigated the influence of the mode of transmembrane pressure (Δp_TM_) increase to target level and the deposit layer pressure history on the filtration performance during skim milk MF at temperatures of 10 °C and 50 °C. Two filtration protocols were established: No. 1: Δp_TM_ was set directly to various target values. No. 2: Starting from a low Δp_TM_, we increased and subsequently decreased Δp_TM_ stepwise. The comparison of both protocols tested the effect of the mode of Δp_TM_ increase to target level. The latter protocol alone tested the effect of the deposit layer history with regard to the Δp_TM_. As expected, flux and protein permeation were both found to be functions of the Δp_TM_. Further, both measures were independent of the filtration protocol as long as Δp_TM_ was held at a constant level or, as part of protocol No. 2, Δp_TM_ was increased. Thus, we can state that the mode of Δp_TM_ increase to target level does not affect filtration performance in SWM. We found that after completion of a full cycle of stepping Δp_TM_ up from 0.5 bar to 3.0 bar and back down, flux and deposit layer resistance were not affected by the deposit layer history at 10 °C, but they were at 50 °C. Protein permeation, however, was lower for both 10 °C and 50 °C, when the Δp_TM_ cycle was completed. The processing history had a significant impact on filtration performance due to remaining structural compression effects in the deposited layer, which occur most notably at higher temperatures. Furthermore, temperatures of 50 °C lead to deposit layer aging, which is probably due to an enhanced crosslinking of particles in the deposit layer. Apart from that, we could show that fouling resistance does not directly correlate with protein permeation during skim milk MF using SWM.

## 1. Introduction

Microfiltration (MF) applied for milk protein fractionation has been established as a standard operation in the dairy industry in the last years. Polymeric spiral-wound membranes (SWM) are much less commonly used, but are developing as an emerging alternative to ceramic and polymeric tubular membranes. As shown by Le Berre and Daufin [1] for skim milk MF with ceramic membranes and by Beckman et al. [2], Beckman and Barbano [3], and Zulewska and Barbano [4] for polymeric membranes, flux and whey protein permeation through the membrane are seriously affected by fouling due to protein deposition. It is known that higher levels of transmembrane pressure (Δp_TM_) induce compression of deposited protein layers. However, what remains unclear is whether, or to what extent, the pressure adjustment scheme, i.e., the mode of Δp_TM_ adjustment to target level, affects MF flux and protein permeation. A stepwise increase or decrease of Δp_TM_ creates a different situation in terms of processing history, possibly affecting the deposit’s structural properties. Furthermore, long-term continuous operations (of around 6–24 h) between two membrane cleaning cycles also create aging effects related to the processing history, especially when higher temperatures, i.e., around 50 °C, are compared to lower temperatures, i.e., around 10 °C. It can be expected that structural time-dependent modifications of the deposited layer occur at higher temperatures due to more intense interactions between particles in the deposit layer.

At the moment of changing from water flowing through the clean membrane to milk, the initial flux is high. No deposit has formed on the membrane surface at this stage. Therefore, protein particles, casein micelles and whey proteins, are transported at a high rate toward the MF membrane surface and penetrate partially into the membrane´s still-open pores. Hence, pore blocking and deposit layer formation can occur rapidly [5]. In skim milk filtration at 50 °C using SWM with a pore size of 0.3 µm, Zulewska et al. [4] reported a high casein permeation within the first few minutes of filtration that then decreased quickly. They supposed that internal pore blocking would take place as the initial fouling mechanism prior to deposit layer formation on the membrane´s surface. Since the initial flux is significantly higher than the flux at steady-state conditions [6], mass transfer to the membrane surface is enhanced when the membrane has just been put in operation after the cleaning stage. Beckman and Barbano [3] stated that concentration polarization is distinct at a filtration temperature of 50 °C, resulting in a gel layer formed by the retained proteins, especially at higher concentration factors (CF). These authors also stated that pore blocking would be the main fouling contributor, when the initial deposit layer formation is less pronounced, i.e., at a lower CF. Pore blocking was associated with a lower whey protein permeation as compared to deposit layer formation on the membrane surface. However, the effect of the initial fouling has not been tested experimentally in SWM during MF yet.

The initial fouling should be related to the mode of Δp_TM_ increase to target level. Gésan-Guiziou et al. [7], however, found no difference in flux or protein permeation, irrespective of whether the target Δp_TM_ value was set directly or approached gradually starting from a lower value during ceramic MF of skim milk at 50 °C. In those tests, a concentration of skim milk to CF 2 was carried out prior to the experiment. No explicit conclusion about the influence of the mode of Δp_TM_ increase beginning at the change over from water to skim milk can therefore be drawn. 

Despite the results of Gésan-Guiziou [7], the deposit layer formation history can generally be expected to have an influence on the filtration performance. For an operating temperature of 50 °C, Gésan-Guiziou et al. [8] reported a reduction of flux and protein permeation after one pressure cycle, i.e., increasing and subsequently decreasing the Δp_TM_ stepwise, during ceramic MF. The authors attributed this to irreversible modifications of the deposit layer when a certain flux to wall shear stress ratio was exceeded. 

Higher Δp_TM_ levels were found to induce an irreversible compression of the deposit. Qu et al. [9] reported that casein micelles suspensions form highly compressible layers when the Δp_TM_ exceeds the critical osmotic pressure for gel formation of about 0.35 bar. At this point, casein micelles overlap and fuse to a compact gel [10]. Thus, the porosity of the casein micelles bodies becomes the determining factor to the deposit layer resistance [9]. With increasing Δp_TM_, compression is more pronounced, reducing deposit layer´s porosity [11,12]. Under these conditions, enhanced particle interactions, and thus, crosslinked deposit structures, can be expected, especially at high Δp_TM_ values.

Upon Δp_TM_ release from a higher level, a compressed deposit layer can be expected to relax depending on the stabilizing links between the deposited particles. Qu et al. [9] observed that the filtration resistance reached its initial value after an intermediate pressure application of 0.5 bar Δp_TM_. With increasing Δp_TM_ (2.0 bar and 4.0 bar, respectively) and number of pressure cycles, the recovery of the filtration resistance became increasingly lower. Thus, the alteration of the deposit layer structure seems to be dependent on its Δp_TM_ history. Other works on skim milk ultrafiltration by Steinhauer et al. [13] did not report on a significant increase in irreversible gel formation by proteins in the deposit layer induced by higher levels of Δp_TM_. The operating temperature in this case was 20 °C. With regard to protein interactions, the reactivity of native β-lactoglobulin (β-lg) as contributor to structure creation in deposit layers has to be considered. Steinhauer et al. [14] reported on the formation of β-lg molecule clusters on the casein micelles due to thiol-disulfide reactions, even below denaturation temperature. The interpretation was that due to the close vicinity of proteins in the concentrated deposit layer, the reactivity of β-lg and casein micelle surfaces is high enough to induce cluster formation. Casein micelles lose their steric surface stabilization and the larger clusters then form a more porous deposit layer. This effect was not seen by Qu et al. [9], because they filtered only casein micelles. 

Apart from the pressure application mode, temperature influences the filtration performance, deposit layer formation, and its behavior upon pressure increase. A release of β-casein from the micelle into the serum phase is reported at low temperatures, which increases casein permeation [15]. Besides that, the flux is known to be lower at lower temperatures [16]. This is due to the higher viscosity of the permeate. However, during UF, Ng et al. [17] reported a lower rate of time dependent flux reduction at lower temperatures. While a significant reduction of the flux with increasing filtration time was observed at a filtration temperature of 50 °C, the flux at 10 °C stayed constant. The constant flux at low temperatures was also reported by Jarto et al. [18] during polymeric MF of concentrated skim milk (CF 2) at 13 °C. Ng et al. [17] stated that the temperature dependent reduction of flux during UF is caused by irreversible fouling due to increased deposition of α-lactalbumin (α-la) and by the deposition of β-lg. The β-lg deposition was only present at 50 °C. Even at lower temperatures starting at 40 °C, β-lg exposes its free thiol group, which is buried inside the molecular structure [19]. This is of particular interest, especially when longer-term operations are considered with reaction times of several hours as it is the case with proteins deposited in layers adhered to the membrane surface. The free thiol group is then expected to be able participate in thiol-disulfide reactions leading to a structural consolidation of the deposited protein layers and thus to flux decline [20]. The formation of crosslinked aggregates and intensified fouling behavior due to thiol-disulfide exchange reactions was also reported with bovine serum albumin (BSA), which possesses a free thiol group as well [21]. The progressive reduction of flux [22] and protein permeation [3], which was reported for polymeric MF of skim milk at high temperatures but not at low temperatures [18], could therefore be due to enhanced fouling driven by thiol-disulfide reactions. Aside from that, other effects, such as an increased stability of the hairy layer of the casein micelle and the lower tendency for hydrophobic interactions at low temperatures [23], might influence the deposit layer formation. The precipitation of minerals (in particular calcium phosphate), however, was not reported to be a distinct contributor to fouling during skim milk filtration at either high or low temperatures [17].

The extent to which the compression process during crossflow MF of skim milk using SWM is reversible at low and high temperatures remains unclear. Further, it is unknown whether flux and protein permeation during MF using SWM are equally affected by decompression of the deposited layer at lower levels of Δp_TM_, following higher levels.

From the results reported in literature so far, it seems unclear whether the deposit layer history with regard to the mode of Δp_TM_ increase, i.e., stepwise or directly to target level, deposit layer aging, and structural alterations has a distinct influence on the MF performance of SWMs. Therefore, the purpose of this study was to assess the effects of the mode of Δp_TM_ increase to target level on the filtration performance during MF with SWM. Further, the objective was to unravel the effect of the deposit layer Δp_TM_ history on the MF for milk protein fractionation using polymeric SWM at low and high filtration temperatures.

The approach we applied was a combination of two filtration protocols:

- Setting Δp_TM_ to various target levels and keeping it constant over the entire experiment (No. 1)
- Increasing and subsequently decreasing Δp_TM_ stepwise (No. 2)

By mode No. 1, time dependent changes in filtration behavior and deposit layer formation could be investigated [24]. Furthermore, the intensity of initial fouling can also be varied. To analyze the influence of the way to approach the target Δp_TM_, i.e., the mode of Δp_TM_ increase to target level, on filtration performance, a stepwise increase of Δp_TM_ has to be performed as an additional set of experiments. In addition, the results obtained by protocol No. 2 showed the influence of the deposit layer history on the filtration performance. Since compression is expected to alter the deposit layer structure, additional information about the effects on flux and protein permeation reduction should become clear.

The results of this study show that the mode of Δp_TM_ increase to target level does not affect filtration performance during polymeric MF. After compression, on the other hand, the structure of the deposit layer is altered irreversibly. This reduces protein permeation independently of the temperature. However, the flux is just affected at high temperatures. Therefore, the deposit layer crosslinking must be higher at elevated temperatures.

## 2. Materials and Methods

### 2.1. Filtration Fluid Skim Milk and Analyses

Molkerei Weihenstephan (Freising, Germany) provided pasteurized skim milk (74 °C, 28 s), which was stored at 4 °C before the tests (up to 4 days). The contents of casein and of the major whey proteins β-lg and α-la were analyzed using reversed phase high performance liquid chromatography (RP-HPLC) as reported by Dumpler et al. [25].

Viscosities of water and MF permeate were measured with an Anton Paar MCR 302 rheometer (Anton Paar, Graz, Austria) using the double-gap geometry. The dynamic viscosities at 10 °C were 1.28 mPa·s and 1.62 mPa·s for water and MF permeate, respectively. At 50 °C, MF permeate viscosity was 0.74 mPa·s. In all conducted experiments, MF permeate viscosity was independent of the Δp_TM_.

### 2.2. Membrane and Filtration Pilot Plant

In this study, a filtration pilot plant for SWM (SIMA-tec, Schwalmtal, Germany) was used. Filtration trials were conducted with a 0.1-µm spiral-wound polyvinylidene fluoride (PVDF) membrane (46 mil diamond-shaped spacer) manufactured by Synder Filtration (Vacaville, CA, USA). The SWM had a diameter of 0.16 m, a length of 0.96 m, and an active filtration area of 16.35 m^2^.

#### 2.2.1. Pilot Filtration Plant and Experimental Design

**Figure 1:**
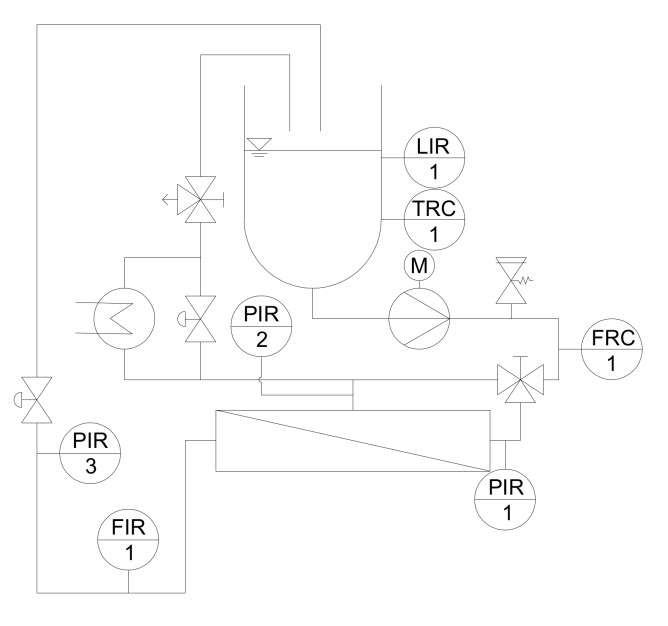
Simplified P&I diagram of the pilot plant (LIR: Level indicator recorder; TRC: Temperature recorder controller; PIR: Pressure indicator recorder; FRC: Flow recorder controller; FIR: Flow indicator recorder).

The pilot plant is shown in a simplified piping and instrumentation (P&I) diagram in Figure 1.

A 1.3 m-long pressure housing contained the SWM and was equipped with two cartwheel-shaped anti-telescoping devices. A multistage centrifugal pump drove a feed volume flow of 18 m^3^·h^−1^. Temperature was controlled by a heat exchanger installed in the retentate stream and ServiceLab 12 (ServiceLab, Neu-Ulm, Germany) was used to record filtration data. At low Δp_TM_ values, the permeate stream was throttled, i.e., a permeate counter pressure was applied without throttling the retentate stream. When the permeate counter pressure was completely released, the retentate stream was throttled to reach higher Δp_TM_ values. The pressure transducers were positioned directly in front of and behind the SWM. Therefore, no significant influence of their position on the calculation of the mean Δp_TM_ is to be expected. Δp_TM_ was calculated by Equation 1 from the pressure at the membrane inlet p_i_, the pressure at the membrane outlet p_i+1_, and permeate pressure p_p_.

(1) $$\Delta p_{\mathrm{TM}}\;=\;\frac{p_{i}+p_{i+1}}{2}\;-\;p_{p}$$

#### 2.2.2. Start-Up Procedure and Experimental Design

Prior to filtration, the membrane was conditioned with caustic Ultrasil 69 (0.4% *v*/*v*, Ecolab Deutschland, Monheim am Rhein, Germany) at 50 °C for 20 min to charge the membrane negatively. Then, 150 L of skim milk (native pH of 6.8 at 10 °C) were heated from 4 °C to the process temperatures of 10 °C and 50 °C, respectively. The filtration plant was flushed with 50 L skim milk without permeate production (permeate valve closed). Pressure was adjusted to the target Δp_TM_ and the permeate and retentate streams were recirculated. After the start-up procedure, flux was measured continuously and filtration tests were run with one of the following filtration protocols:

- Stepwise increasing and subsequently stepwise decreasing Δp_TM_
- Setting Δp_TM_ directly to target level and keeping it constant during the experiment

#### 2.2.3. Stepwise Increasing and Subsequently Stepwise Decreasing Δp_TM_

After adjusting the Δp_TM_ to 0.5 bar, the Δp_TM_ was held constant for 40 min with the objective to achieve steady-state filtration conditions. Subsequently, the Δp_TM_ was increased gradually in steps of 0.5 bar every 30 min to 3.0 bar, i.e., Δp_TM_ values of 0.5, 1.0, 1.5, 2.0, 2.5, and 3.0 bar were tested. In some cases, the Δp_TM_ was then reduced to 0.5 bar, again in steps. Permeate samples were taken at the end of each pressure step and the retentate was sampled from the feed tank after 40 min (duplicate) and at the end of the experiment (single).

#### 2.2.4. Setting Δp_TM_ Directly to Target Level and Keeping It Constant during the Experiment

As a second filtration protocol, experiments were conducted with setting the Δp_TM_ directly to target level and keeping it constant for 120 min, 480 min (8 h) for 50 °C, or 1440 min (24 h) for 10 °C to investigate whether the steady-state filtration would endure over periods common in the dairy industry. Furthermore, the effect of the mode of Δp_TM_ increase to target level on filtration performance was investigated. Permeate samples were taken throughout the filtration experiment and retentate samples were taken after 40 min (duplicate) and at the end of the experiment (single).

#### 2.2.5. Cleaning Procedure

Following all filtration experiments, the membrane was flushed with deionized water close to the filtration temperature of the previous experiment until the retentate was optically clean. Combined caustic (0.8% *v*/*v* Ultrasil 69, Ecolab Deutschland) and enzymatic (0.3% *v*/*v* Ultrasil 67, Ecolab Deutschland) cleaning was performed at 50 °C for 40 min. Afterwards, an acidic cleaning step with 0.4% *v*/*v* Ultrasil 75 (Ecolab Deutschland) was carried out for 30 min at 50 °C. Both cleaning steps were adapted from the producer´s recommendations. The water flux of the SWM was measured prior to each experiment to verify a sufficient cleaning.

### 2.3. Calculations

Protein permeation was calculated with Equation 2 from the concentration of the target protein in the permeate c_p_ and the protein concentration in the retentate after 40 min c_r, 40 min_.

(2) $$P\;=\;\frac{c_{p}}{c_{r,40\;\min}}\times 100\%$$

Filtration resistances were calculated using Equation (3). In case of membrane resistance R_m_, water was used as the filtration fluid. Therefore, the fouling resistance R_f_ was negligible. R_m_ was 2.05 × 10^12^ ± 5.33 × 10^9^ m^−1^ and stayed constant throughout the experiments. When measuring R_f_, milk was the filtration fluid and the membrane resistance was subtracted from the total resistance (R_f_ + R_m_). In all calculations, η is the permeate´s dynamic viscosity.

(3) $$J\;=\;\frac{\Delta p_{\mathrm{TM}}}{\eta \;\times \;\left(R_{f}+{\;R}_{m}\right)}$$

### 2.4. Data Regression and Statistical Analysis

Data was plotted using OriginPro 2017G (OriginLab Corporation, Northampton, MA, USA). Error bars represent the standard deviation found in two separate experiments conducted with milk from different lots.

## 3. Results and Discussion

### 3.1. Effect of the Mode of Δp_TM_ Increase to Target Level on Flux of SWM

In this study, the effect of the mode of Δp_TM_ increase on deposit layer formation and filtration performance of SWM during skim milk MF was investigated. In particular, it was analyzed, whether a stepwise increase of Δp_TM_ alters the filtration performance compared setting Δp_TM_ directly to a higher initial value and keeping it constant throughout the experiment. This was investigated with low and high temperatures, i.e., 10 °C and 50 °C.

#### 3.1.1. Influence of Temperature and Initial Δp_TM_ on Steady-state Conditions of Flux

**Figure 2:**
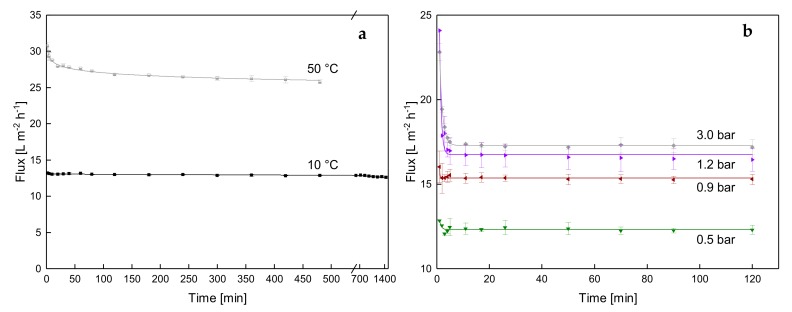
Flux as a function of time at different temperatures at the Δp_TM_ of 0.5 bar (**a**) and at different Δp_TM_ values at 10 °C (**b**).

First, it was investigated how the flux evolves with filtration time and how much time is needed to reach steady-state conditions with a constant Δp_TM_. This was to ensure that the effect of the intensity of deposit layer formation on filtration performance is not obscured by incomplete equilibration at phase-interface between the deposit layer and the bulk. Figure 2 shows the flux as a function of filtration time at different temperatures (a) and different Δp_TM_ values (b).

The flux decreased distinctly in the first 5 min. After the initial decrease, a steady-state flux of 13.0 L·m^−2^·h^−1^ was achieved at 10 °C and 0.5 bar, which could be maintained for 24 h. The time dependent flux decrease was about 0.02 L·m^−2^·h^−2^. Although, the flux at 50 °C was about twice as high (27.8 L·m^−2^·h^−1^ at 40 min) as that at 10 °C, it constantly decreased by the mean rate of 0.32 L·m^−2^·h^−2^ between 60 min and the end of the filtration at 480 min. Thus, no complete steady-state conditions could be obtained at 50 °C, while at 10 °C, the flux remained stable throughout.

In the initial filtration phase, the high flux values result from the absence of a deposit layer on the membrane surface. As soon as the Δp_TM_ is adjusted, the filtrate stream convectively transports proteins to the membrane surface, where they accumulate due to size-based retention effects and form a deposit layer [5]. The deposit layer causes a reduction of the effective Δp_TM_ on the membrane surface [12]. Thus, the presence of a deposit layer reduces the flux. Besides that, single proteins can cause pore blocking inside the membrane pores or at the pore inlets [5]. Hence, those pores do not take part in the filtration anymore. As a consequence of both fouling mechanisms, the filtration resistance increases and flux decreases. This process only lasts for a certain period, after which the deposit layer formation reaches an equilibrium at low temperatures (in our case after 5 min). Jarto et al. [18] reported a similar behavior in experiments with polymeric membranes at 13 °C. The main contribution to membrane fouling occurs within the first few minutes. For short filtration times and at higher CF levels, a slightly decreasing and a constant flux at temperatures around 50 °C [22] and 10 °C [18], respectively, was reported for polymeric SWM. This shows that deposit layer aging is a temperature-related mechanism. This could be explained as follows. According to Iametti et al. [19], the free thiol group of β-lg becomes exposed and accessible for intermolecular reactions with other proteins at 50 °C. Thus, β-lg accumulates in the deposit structure [17] and can induce other proteins to accumulate in the deposit layer. Since the reactive thiol group of β-lg is buried inside the molecules at low temperature [19], this reaction is less likely to happen and no β-lg related aging of the deposit layer is to be expected at 10 °C. Disregarding β-lg, BSA is known to have a free thiol group, which could also contribute to thiol disulfide reactions [21]. Furthermore, it was reported that hydrophobic interactions are more enhanced at higher temperatures and the hairy layer around the casein micelles is less stable [23]. This could also cause a tightening of the deposit structure, which then reduces the flux. It is to be evaluated in further studies, which mechanism is mainly responsible for the temperature-related deposit layer aging.

Apart from the temperature, the effect of the Δp_TM_ on flux progression was investigated at 10 °C (Figure 2b). We observed that the initial flux reduction was more pronounced with higher Δp_TM_ values. At the Δp_TM_ of 3.0 bar, flux at 1 min was 22.8 L·m^−2^·h^−1^ and it decreased to 17.4 L·m^−2^·h^−1^ by 11 min. After the initial phase, the flux stayed effectively constant and reached 17.2 L·m^−2^·h^−1^ at 120 min. The increase in fouling resistance is more distinct at elevated Δp_TM_, which can be attributed to more intense transport of proteins toward the membrane surface. However, Δp_TM_ had no influence on the flux equilibration time until a steady state was reached. Independently of Δp_TM_, a holding time of 5 min was sufficient to achieve steady-state conditions at low temperatures.

#### 3.1.2. Influence of a Gradual Increase of Δp_TM_ on Steady-state Conditions of Flux

**Figure 3:**
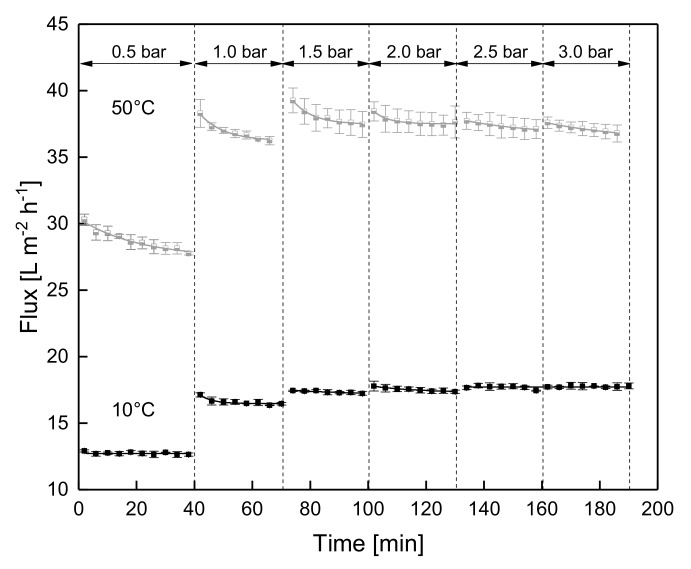
Flux as a function of filtration time during the stepwise increase of Δp_TM_ at 10 °C and 50 °C.

Based on the results shown above, steady-state conditions are expected to be reached at low temperatures soon after each step of Δp_TM_ increase as well. For temperatures of about 50 °C, a progressing deposit layer aging is to be expected in addition to the impact of the Δp_TM_ increase. Figure 3 plots the flux as a function of the filtration time during the stepwise increase of Δp_TM_ (step height 0.5 bar) from 0.5 bar to 3.0 bar at 10 °C and 50 °C. As already seen in Figure 2, the flux decreases significantly during the first few minutes due to deposit layer formation. 

At 10 °C, the flux stayed constant and steady-state conditions were reached. A subsequent increase in Δp_TM_, however, increases flux according to Darcy´s law. Thereby, a new equilibrium was established at the interface between deposited particles and the bulk phase, as already reported by Field et al. [26]. However, the decline in flux was less distinct compared to starting at a higher initial Δp_TM_ (Figure 2), as the membrane surface was already fouled to a certain extent. The process of additional deposit layer formation takes approximately 5 min as the flux decreased from 17.1 L·m^−2^·h^−1^ directly after the Δp_TM_ was adjusted to 1.0 bar to 16.5 L·m^−2^·h^−1^, once the new steady state was reached. At 1.5 bar, limiting flux conditions were obtained. Further increase in Δp_TM_ did not cause a significant increase in flux [27]. This can be attributed to compression and deformation of the proteins in the deposit layer and a more intense gelation [9,11,28]. It has to be noted that no reduction of the flux due to deposit layer aging could be observed with low temperatures. This is in contrast to the observations at 50 °C.

As expected from Figure 2, no steady state was reached at 50 °C. An increase in Δp_TM_ enhanced the flux, until the limiting flux was reached at about 37 L·m^−2^·h^−1^. Independently of the Δp_TM_, the filtration was overlaid by the temperature induced deposit layer aging. 

#### 3.1.3. Influence of the Mode of Pressure Adjustment on Steady-state Flux

**Figure 4:**
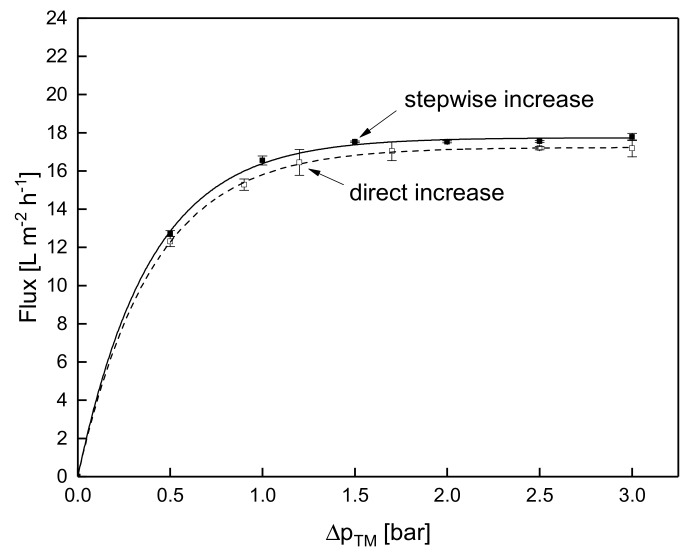
Comparison of flux after a stepwise (filled symbols and solid line) and a direct Δp_TM_ increase to target level (blank symbols and dashed line) at 10 °C.

Whether the flux levels of both filtration protocols were similar was only assessed at 10 °C due to the aging effect at 50 °C. This is of interest, as it indicates whether the mode of Δp_TM_ increase affects the flux. Figure 4 shows the flux values during the stepwise increase of Δp_TM_ at 10 °C along with those measured during filtration experiments conducted at a constant Δp_TM_ value for 120 min. In other words, Figure 4 shows the flux for the two different modes of Δp_TM_ increase to target level. The flux values measured in steady-state conditions are equal. Flux increased from 12.7 L·m^−2^·h^−1^ and 12.3 L·m^−2^·h^−1^ to 17.8 L·m^−2^·h^−1^ and 17.2 L·m^−2^·h^−1^ for the direct and the stepwise pressure increase to target level, respectively, when increasing the Δp_TM_ from 0.5 bar to 3.0 bar. Regarding flux, a deposit layer formed at a lower Δp_TM_ does not affect the subsequent flux at higher Δp_TM_ values. This means that flux at low filtration temperatures does not depend on the mode of Δp_TM_ increase. In contrast to low temperatures, the rate of pressure adjustment is expected to slightly affect the flux at high temperatures. This is due to the fact that the flux at 50 °C was seen to be time dependent.

In contrast to that, Meyer et al. [29] during ceramic UF and Gésan-Guiziou et al. [7] during ceramic MF observed no effect of the filtration protocol at 50 °C. Both studies increased Δp_TM_ stepwise and compared the results against filtration experiments, in which the Δp_TM_ was directly set to the target value. Thus, the filtration performance of a pre-fouled membrane was compared to that of a clean membrane. Both studies found that filtration performance at 50 °C was also independent of the fouling layer generated at lower Δp_TM_. This is probably due to the fact that significantly longer equilibration at the initial Δp_TM_ step was carried (90 min by Meyer et al. [29] and a concentration to CF 2 plus an additional 45 min by Gésan-Guiziou et al. [7]). Since the rate of flux decrease declines with the filtration time, a longer equilibration time leads to a more constant flux at 50 °C. Thus, deposit layer aging effects have a less pronounced impact. In relation to our experiments, it can be expected that no effect of the pressure adjustment mode would be observable at 50 °C after a longer equilibration time.

### 3.2. Effect of the Deposit Layer History on Flux of SWM

**Figure 5:**
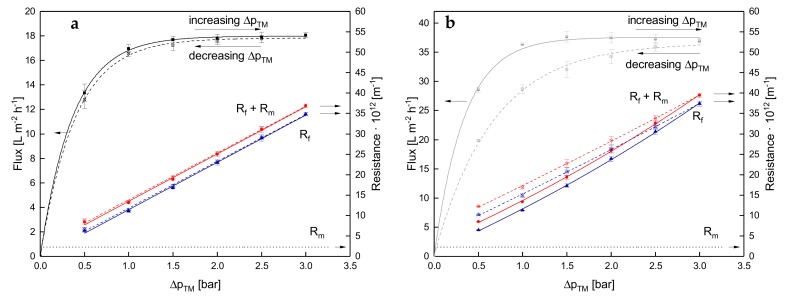
Flux and filtration resistances as a function of Δp_TM_ for one cycle of a stepwise increase of Δp_TM_ (filled symbols and solid lines) and the subsequent stepwise reduction of Δp_TM_ (crossed symbols and dashed lines) at 10 °C (**a**) and 50 °C (**b**). Error bars represent the standard deviation of the flux and resistance values of two individual experiments with milk from two different lots.

The results presented above show that the mode of Δp_TM_ increase to target level did not influence flux or fouling resistance during skim milk MF as long as steady-state conditions were reached. Furthermore, a previously formed deposit layer does not affect the subsequent flux at higher Δp_TM_ apart from potential aging effects. It is further of interest, whether deposit layers originating from higher Δp_TM_ values show the same filtration behavior. Figure 5 plots the flux and filtration resistances as a function of Δp_TM_ during a stepwise increase and the subsequent stepwise reduction of Δp_TM_, i.e., one full pressure cycle.

The subsequent reduction of Δp_TM_ does not affect the dependence of flux on Δp_TM_ at 10 °C (Figure 5a). At 0.5 bar, the flux was 12.8 L·m^−2^·h^−1^ and 12.3 L·m^−2^·h^−1^ prior to and after a temporary increase of Δp_TM_, respectively. As seen in Figure 2, no aging effects could be detected, since the flux was independent of filtration time after the initial decrease in flux due to initial fouling. Compression of the deposit layer caused by Δp_TM_, therefore, seems to be completely reversible. In this filtration protocol, the deposit layer resistance is independent of the Δp_TM_ history as it reached the same level after a temporary increase of Δp_TM_ (6.27 × 10^12^ m^−1^ and 6.63 × 10^12^ m^−1^ at 0.5 bar Δp_TM_ prior to and after the temporary increase of Δp_TM_, respectively).

In contrast to 10 °C, flux at 50 °C showed a distinct hysteresis comparing ascending and descending Δp_TM_. At 0.5 bar, the flux values of 27.8 L·m^−2^·h^−1^ and 20.0 L·m^−2^·h^−1^ were found before and after the pressure cycle, respectively. The results at 50 °C go along with the findings of Gésan-Guiziou et al. [8]. The authors processed skim milk (CF 2.0) with a 0.1-µm ceramic tubular membrane at 50 °C. They increased the Δp_TM_ from 0.0 bar to 1.0 bar and then decreased it to 0.0 bar over about 120 min. At about 0.3 bar, the flux was 75 L·m^−2^·h^−1^ and 65 L·m^−2^·h^−1^ during the increasing and decreasing phases, respectively. They attributed the hysteresis to the accumulation of deposits on the membrane and their irreversible modification after the ratio of flux to wall shear stress exceeded a critical value. Exceeding this critical ratio could be the cause for the differences in our study with SWM as well, since flux values are significantly lower at 10 °C compared to 50 °C. Besides that, our results give evidence that the phenomenon is temperature related. During compression of the deposit layer at elevated Δp_TM_ values, proteins are brought together closely, increasing the chance of crosslinking. An increased crosslinking would, independently of the underlying mechanism, stabilize the compressed structure resulting from high Δp_TM_ values. After pressure release, the compressed structure seems to be partly preserved at high temperatures reducing the flux after an intermediate pressure increase. The alteration of the deposit layer properties seems to be less pronounced at 10 °C, as the deposit layer resistance returns to its initial value. Hence, flux at low temperatures is obviously independent of the Δp_TM_ history.

### 3.3. Effects on Protein Permeation in SWM and the Development of Steady-state Filtration Conditions

#### 3.3.1. Influence of the Temperature and Protein Size on the Development of Steady-state Protein Permeation

**Figure 6:**
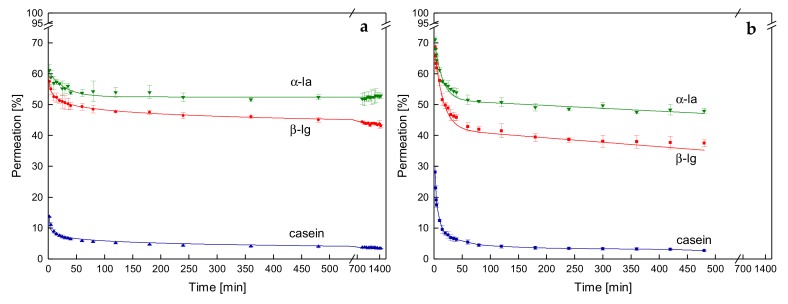
Permeation of different-sized proteins as a function of time at the Δp_TM_ of 0.5 bar and 10 °C (**a**) or 50 °C (**b**).

The flux was shown to be independent of the mode of Δp_TM_ increase to target level and the deposit layer history at 10 °C but not at 50 °C. However, flux alone is not sufficient to fully characterize filtration performance during skim milk protein fractionation. Protein permeation must also be considered. Figure 6 shows the permeation of the major whey proteins α-la and β-lg along with casein as a function of time at the Δp_TM_ of 0.5 bar for 10 °C (a) and 50 °C (b). The sizes of α-la, β-lg, and casein micelles are 2.29 nm, 4.19 nm, and 182 nm, respectively [30]. Regardless of protein size and temperature, protein permeation drops most intensely within the first minutes.

At 10 °C and 0.5 bar, permeation of β-lg decreased from 69% at 2 min to 63% at 5 min (Figure 6a). Fouling mostly occurs during the initial phase of filtration due to initial pore blocking and deposit layer formation, as also observed with the flux measurements. Proteins are convectively transported through the membrane pores by the filtrate stream. Thus, proteins are among other factors such as charge primarily retained depending on their size as also observed by Heidebrecht et al. [31]. In the present study, this could be confirmed as the permeation of α-la, β-lg, and casein at 120 min and 0.5 bar was 65%, 59%, and 5%, respectively. This corresponds to a protein content in the permeate of 0.78 g·L^−1^, 2.36 g·L^−1^, and 1.45 g·L^−1^, respectively. The protein size did not have any effect on the equilibration time, but it was determined by the formation of the fouling layer per se. Due to the deposition of particles and the gel layer formation, the membrane apparently becomes less permeable for proteins. In MF, the deposit layer is known to act as a secondary selective layer on the membrane [2]. The deposit layer even takes precedence over the properties of the membrane. Thus, after initial fouling, alterations in filtration performance are mainly caused by alterations in the deposit layer. Since the protein permeation stays rather constant after an initial drop (β-lg permeation reduction of 1.2% h^−1^ between 40 min and 180 min at 10 °C), no decisive alterations in the deposit layer happen at low temperatures.

At 50 °C, however, the reduction in permeation is more intense and the duration of the steep decline phase is longer. Furthermore, after the initial steep decrease, protein permeation continues to descend at a mean rate of 2.8% h^−1^ between 40 min to 180 min. This can again be attributed to continuing temperature-induced aging in the deposit layer. Furthermore, the permeation of casein seems rather high for a 0.1-µm membrane (up to 30% at the beginning of the filtration). However, it is known that polymeric membranes have a wide pore size distribution. Thus, the passage of caseins through the pores bigger than 0.1 µm occurs. This effect was also observed by Zulewska et al. [22] during filtration using SWM. After the deposit layer formation, caseins are held back by the layer, which acts as a secondary filtration membrane. 

After the severe decline of protein permeation due to initial fouling, the permeation of casein was higher for the lower temperature. This was due to the release of β-casein from the micelle and its transition into the serum phase. Thus, β-casein permeated along with the whey proteins, which was previously reported [15]. Furthermore, we observed α-la and β-lg permeations to be slightly higher at 10 °C compared to 50 °C. This is another indication that the deposit layer structure at higher temperatures becomes more compact and crosslinked, respectively.

#### 3.3.2. Influence of the Δp_TM_ on the Development of Steady-state Protein Permeation

**Figure 7:**
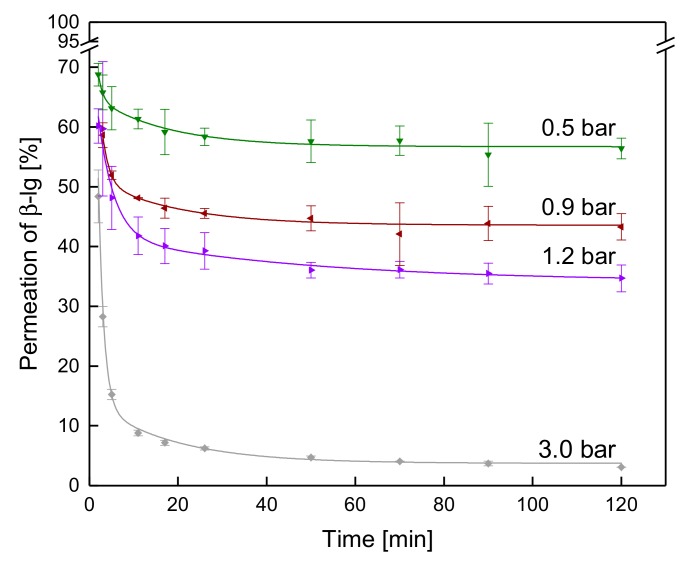
Permeation of β-lg as a function of time at different Δp_TM_ values at 10 °C.

Besides the effect of protein size, the Δp_TM_ is known to have a significant influence on protein permeation in MF. Figure 7 shows the permeation of β-lg at different values of Δp_TM_ as a function of the filtration time at 10 °C.

The permeation of β-lg decreases most pronounced within the first 5 min regardless of Δp_TM_. However, the extent of the decrease in protein permeation increases with the Δp_TM_. At 0.5 bar and 3.0 bar, permeation was reduced by 6 and 33 percentage points, respectively, between 2 min and 5 min after the filtration began. This confirms that deposit layer formation is more intense at higher values of Δp_TM_. The compressive forces on the deposit layer are more pronounced at elevated Δp_TM_. Since the deposit is mostly formed by compressible casein micelles as shown by Steinhauer et al. [14], increasing Δp_TM_ alters deposited casein micelles from a spherical to an ellipsoidal-deformed shape [28]. Furthermore, a more intense compaction and dewatering of the micelles occurs [32]. Therefore, the filtration performance is dominated by the intrinsic structure of the casein micelles, which becomes increasingly more compact [9]. Due to the denser deposit layer structure, the reduction of protein permeation within the first minutes is more distinct at higher Δp_TM_. Our results confirm findings of Heidebrecht et al. [31], who also observed an influence of the ratio of protein to pore size during ceramic microfiltration of skim milk. In case of MF using SWM, the apparent deposit layer pore size seems to be the determining factor. Regardless of the origin of the retaining medium, the ratio of flow channel size to protein size determines protein permeation distinctly.

#### 3.3.3. Influence of a Gradual Increase of Δp_TM_ on the Development of Steady-state Protein Permeation

**Figure 8:**
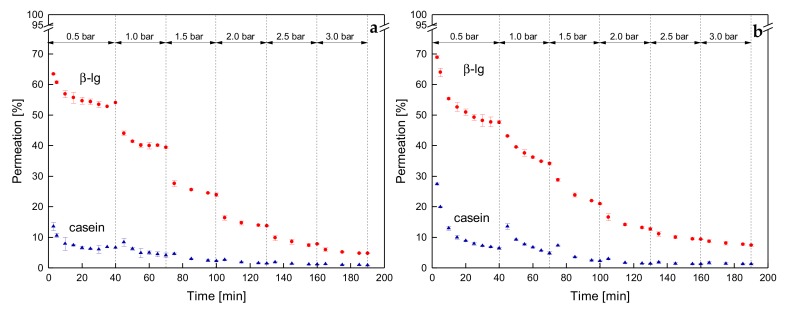
Protein permeation of β-lg (circles) and casein (triangles) as a function of time for stepwise increasing Δp_TM_ at 10 °C (**a**) and 50 °C (**b**).

Besides the influence of the filtration duration on the protein permeation, the influence of a gradual increase of the Δp_TM_ in steps on the protein permeation is of particular interest (Figure 8). Protein permeation decreased significantly during the first 5 min of filtration. At 10 °C, it became effectively constant after 30 min. An increase of Δp_TM_ affects the equilibrium at the membrane surface. After the Δp_TM_ was increased to 1.0 bar, permeation of β-lg dropped from 44% at 5 min to 40% at 15 min after the Δp_TM_ adjustment. 15 min after the Δp_TM_ adjustment, protein permeation remained constant regardless of the Δp_TM_. Since a deposit layer has already formed, equilibration at the interface between deposit layer and the bulk phase occurred faster than it did with a clean membrane. Therefore, it is meaningful that the step duration is longer for the initial step. Based on our findings for 10 °C, a step duration of 40 min in the first step and 30 min in the following steps leads to steady-state conditions.

In contrast to that, steady-state conditions were not completely achieved at 50 °C. Although, the decrease in protein permeation levels off, it is observable that the respective 40 min and 30 min equilibration times are rather short. An increased equilibration time would lead to a more constant protein permeation although aging effects would still be more pronounced than at 10 °C. The more intense decrease, the longer duration until protein permeation levels off, and the lower protein permeation values at 50 °C compared to 10 °C endorse the assumption that a more compact and probably interconnected deposit layer forms at higher temperatures.

#### 3.3.4. Influence of the Mode of Pressure Adjustment on Protein Permeation

**Figure 9:**
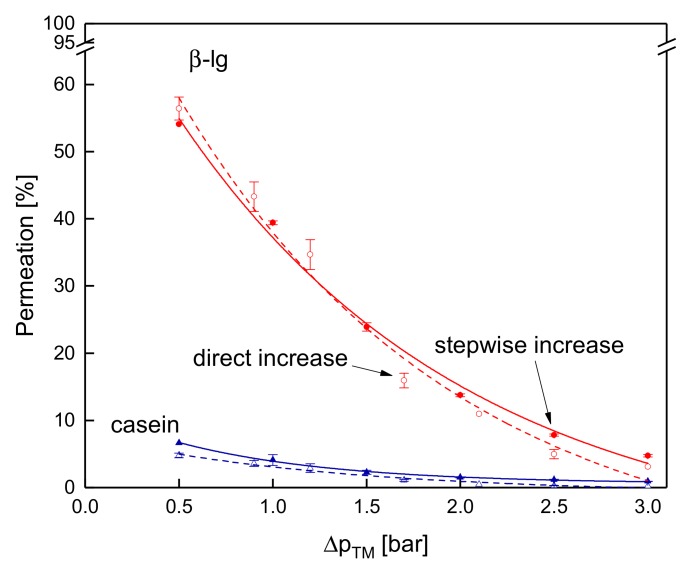
Permeation of β-lg (circles) and casein (triangles) at 10 °C measured with the stepwise increase (filled symbols and solid lines) and direct pressure increase to target level (blank symbols and dashed lines).

Steady-state conditions were reached with both filtration protocols. However, regarding temperature, a steady state was only reached at 10 °C due to the lack of intense deposit layer aging. Again, it is of interest whether the mode of Δp_TM_ increase to target level affects filtration performance, this time with regard to protein permeation. Figure 9 plots the protein permeation at 10 °C obtained with a stepwise increase of the Δp_TM_ and by setting the Δp_TM_ to a higher value initially. It was observed that no influence of the filtration protocol and thus of the mode of Δp_TM_ increase on protein permeation is detectable. At the Δp_TM_ of 0.5 bar, the permeation of β-lg was 54% for the stepwise increase and was 56% for the direct Δp_TM_ increase to target level. The permeation of casein was 7% and 5%, respectively. Although the mode of Δp_TM_ increase differs, the filtration performance is not significantly affected, as can be seen with the protein permeation (Figure 9) and the flux (Figure 4).

Furthermore, it was found that the deposit layer formed at a lower Δp_TM_ does not affect the subsequent protein permeation at higher Δp_TM_ values. Since filtration performance was found to be independent of the initial deposit layer formation, a higher Δp_TM_ only induces more intense fouling. However, the structure of the deposit layer obtained by the stepwise increase is likely to be similar to the deposit layer obtained by the rapid approach to the target Δp_TM_, since the filtration performance is the same.

These findings are presumably transferable to the filtration with SWM at higher temperatures, since Gésan-Guiziou et al. [7] observed the protein permeation to be independent of the filtration protocol during ceramic MF (0.1 µm) at 50 °C. Filtration performance was the same when the Δp_TM_ was increased stepwise every 15 min or was increased directly to the target Δp_TM_. However, they had a longer equilibration time at the first pressure step than we used in our experiments. As seen in Figure 6b, the rate, at which the protein permeation declined, decreased with the filtration duration. Thus, a longer equilibration time would result in a more constant filtration. The impact of deposit layer aging on protein permeation would therefore become virtually negligible.

### 3.4. Influence of the Deposit Layer History on Protein Permeation in SWM

**Figure 10:**
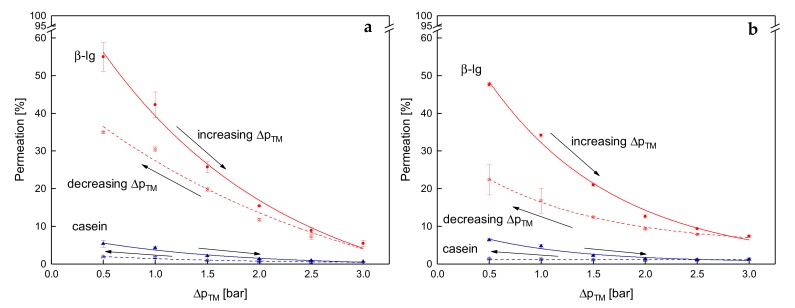
Permeation of β-lg (circles) and casein (triangles) as a function of Δp_TM_ for one cycle of a stepwise increase of Δp_TM_ (filled symbols and solid lines) and the subsequent stepwise reduction of Δp_TM_ (blank symbols and dashed lines) at 10 °C (**a**) and 50 °C (**b**). Error bars represent the standard deviation of the permeation values of two individual experiments with milk from two different lots.

With regard to flux, an intermediate pressure increase had a negative effect on the filtration at 50 °C but not at 10 °C. Contrary to that, the stepwise increase and decrease of Δp_TM_ reduces protein permeation independently of the temperature (Figure 10).

At 0.5 bar and 10 °C, the permeation of β-lg was 56% before the stepwise Δp_TM_ increase and was 35% after the stepwise decrease. The casein permeations were 7% and 5%. This distinct difference in protein permeation cannot be explained by aging effects (total filtration time of 340 min), as the decrease in β-lg permeation at 0.5 bar was lower than 4 percentage points between 40 min and 340 min (compare Figure 6a). At 50 °C, the same trends are observable, however, the extent of changes in the protein permeation are higher. The β-lg permeation decreased from 48% to 22% before and after the pressure cycle. The corresponding time dependent reduction was again not responsible for the decline after the pressure cycle as the β-lg permeation after 340 min at 0.5 bar was 38% (Figure 6b). 

Upon pressure increase, the casein deposit layer becomes compressed. Thus, a transition from a porous structure of the micelles to a gel-like structure occurs [9]. With increasing compression, a shrinking and dewatering of the micelles was reported [32]. Thus, the micelles’ porosity decreases. As discussed above, at the compressed state, an alteration of the deposit layer structure occurs. This might be attributed to an increased crosslinking in the deposit layer resulting in an enforcement of the structure, which is formed at high Δp_TM_. Upon pressure release, the existing crosslinks remain, resulting in a denser deposit structure. The difference between low and high filtration temperatures on the structure of the deposit layer can again be attributed to more intense interactions at higher temperatures. However, the mechanisms, which cause the more compact deposit layer have to be further investigated.

Apart from the dependence of protein permeation on the deposit layer pressure history at 10 °C and 50 °C, the results show that protein permeation is not directly correlated with the fouling resistance. Otherwise, lower protein permeation would be accompanied by higher fouling resistance at 10 °C. Consequently, the reduction in protein permeation is not directly caused by the increase in fouling resistance.

## 4. Conclusions

The study shows that in cases of compressible deposit layers, the mode of Δp_TM_ increase does not affect filtration performance in SWM. In other words, the filtration performance during skim milk MF is independent of the time frame, in which the Δp_TM_ is increased to target level (disregarding aging effects at temperatures about 50 °C).

At higher Δp_TM_, the structure of the casein micelles in the deposit layer becomes denser due to compression. When the Δp_TM_ is reduced afterwards, the compacted structure of the deposit partly remains. Since protein permeation depends on the interaction between pore and protein size [31], filtration performance decreases upon an intermediate pressure increase. Enhanced crosslinking in the deposit layer is likely to be responsible for this; however, the mechanism requires more in-depth study.

Apart from the results on deposit layer formation, it was shown that the filtration performance was practically constant at 10 °C for 24 h. At 50 °C, a progressing slight reduction of the filtration performance was observed. This indicates that the deposit layer aging is temperature-dependent.

Further, the filtration protocol did not affect filtration performance as long as steady-state conditions were attained and the Δp_TM_ was not reduced. Therefore, data acquisition can be carried out with a stepwise increase in Δp_TM_, but not with a decrease or an adjustment at random. In the case of skim milk microfiltration at low temperatures, we propose 40 min for the initial Δp_TM_ step and 30 min for subsequent steps. At 50 °C, longer equilibration times have to be chosen.
